# The Association Between Vitamin E Deficiency and Critically Ill Children With Sepsis and Septic Shock

**DOI:** 10.3389/fnut.2021.648442

**Published:** 2021-06-16

**Authors:** Hongxing Dang, Jing Li, Chengjun Liu, Feng Xu

**Affiliations:** ^1^Department of Pediatric Intensive Care Unit, Ministry of Education Key Laboratory of Child Development and Disorders, Children's Hospital of Chongqing Medical University, Chongqing, China; ^2^National Clinical Research Center for Child Health and Disorders, China International Science and Technology Cooperation Base of Child Development and Critical Disorders, Chongqing, China; ^3^Chongqing Key Laboratory of Child Health and Nutrition, Chongqing, China

**Keywords:** vitamin E, critical ill, sepsis, septic shock, pediatrics

## Abstract

**Background:** Literature is scarce on the assessment of vitamin E status in septic children. We aim to investigate the prevalence of vitamin E deficiency in critically ill children with sepsis and septic shock and its association with clinical features and outcomes.

**Methods:** We compared serum vitamin E status between the confirmed or suspected infection and no infection groups, the sepsis shock and no sepsis shock groups upon pediatric intensive care unit admission. Clinical characteristics were compared in subgroup patients with and without vitamin E deficiency. The association between vitamin E deficiency and septic shock were evaluated using univariate and multivariable methods.

**Results:** 182 critically ill children with confirmed or suspected infection and 114 without infection were enrolled. The incidence of vitamin E deficiency was 30.2% in the infection group and 61.9% in the septic shock subgroup (*P* < 0.001). Thirty-days mortality in critically ill children with vitamin E deficiency was significantly higher than that without vitamin E deficiency (27.3 vs. 14.2%, *P* < 0.05). Vitamin E levels were inversely associated with higher pediatric risk of mortality (*r* = − 0.238, *P* = 0.001) and cardiovascular sequential organ failure assessment (*r* = −0.249, *p* < 0.001) scores in critically ill children with infection. In multivariable logistic regression, vitamin E deficiency showed an independent effect on septic shock (adjusted OR: 6.749, 95%CI: 2.449–18.60, *P* < 0.001).

**Conclusion:** Vitamin E deficiency is highly prevalent in critically ill children with sepsis and contributed to the septic shock.

## Introduction

Sepsis has been defined as a life-threatening clinical syndrome cause of substantial morbidity and mortality (1), that complicates the severe infection and is characterized by the host response to systemic inflammatory response syndrome, oxidative stress imbalance, immune disorder, microcirculatory derangements, and end-organ dysfunction (2). Severe sepsis frequently induces shock and death. The overall burden of illness from pediatric sepsis is high globally, and our recent study revealed the in-hospital mortality rate of severe sepsis reached 18.8% (3).

Inflammation is an essential host response in sepsis, the onset and progression of sepsis upon dysregulation of the normal inflammatory reaction, usually with an increase in both proinflammatory and anti-inflammatory mediators, as well as reactive oxygen species production and the antioxidant protection, initiating a chain of events that leads to widespread tissue injury (4, 5).

Vitamin E is an essential component of the cell membrane and plays a significant role as a lipophilic antioxidant in cellular redox homeostasis. The primary bioactive form of vitamin E is alpha-tocopherol (6), it works as a free radical scavenger, protecting polyunsaturated fatty acids, a major structural component of the cell membranes, from peroxidation (7, 8). In the last decade, promising results from *in vitro* and animal studies suggested a potent antioxidant and anti-inflammation potential of vitamin E and its metabolites (9–11). Besides, vitamin E has also demonstrated other vital functions including improvement of the immune response, inhibition of cell proliferation, regulation of gene expression (12–16).

Vitamin E deficiency is uncommon in healthy adults except in unusual circumstances but is more frequently found in children. Micronutrient inadequacy remains a crucial nutritional problem in Chinese children; surveys of healthy children report that vitamin E intakes are well-below the estimated average requirement (17). Children with severe protein-energy malnutrition are more prone to vitamin E deficiency, likely because they have limited stores and are growing rapidly (18). High incidence of malnutrition in critically ill children, but literature on vitamin E remains scarce, especially there is no available data regarding vitamin E status in children with sepsis and septic shock. Sepsis in children induces a hypercatabolic state, and vitamin E may be consumed in large quantities, so it is mandatory to investigate the statue of vitamin E in this situation.

In the present investigation, we examine whether serum vitamin E level is prospectively associated with severe sepsis and septic shock. Based on vitamin E's antioxidant, anti-inflammation, and other beneficial biological properties, we hypothesize that critical children with sepsis have lower serum vitamin E (α-tocopherol) levels and would experience a higher incidence of severe sepsis and septic shock.

## Methods

### Study Subjects

This prospective observational cohort study was conducted in the pediatric intensive care unit (PICU) and Chongqing Key Laboratory of Child Health and Nutrition in the children's Hospital of Chongqing Medical University of China, a tertiary teaching hospital. The hospital institutional review board approved the study [Approval No.: (2015). Ethics Review (Research) No. (92)]. This study was conducted in compliance with the Declaration of Helsinki and registered at the Clinical Trial Registry (http://www.chictr.org.cn/enIndex.aspx; Registration No.: ChiCTR-OOC-15007152).

### Inclusion and Exclusion Criteria

We screened all consecutive patients admitted to PICU from September 2016 to August 2017. Patients who had an estimated PICU stay of more than 48 h and aged from 1 month to 14 years old were deemed eligible to participate and enrolled within the first 24 h of the PICU admission. Exclusion criteria included patients (1) admitted to PICU for postoperative monitoring, or (2) underwent blood purification, or (3) patients were taking drugs that affect vitamin absorption or metabolism. Before the initiation of study-related procedures, parents or surrogates were informed about the study and provided the written informed consent. This study haven no additional interventions were performed on those children.

### Data Collection

Blood specimens (3 mL each) were obtained from the subjects as early as possible upon PICU admission (always within 12 h), before both treatment and enteral and/or parenteral nutrition. Previously obtained and stored excess laboratory samples were retrieved from the clinical laboratory for some patients if we were not able to obtain consent timely at the time of PICU admission. Each blood specimen was collected in tubes and harvested by centrifugation at 3,000 g for 10 min to obtain 1.5–2 mL of serum, keep in dark, then frozen at −80°C and sent in batches to a nationally accredited third party for the quantitative analysis of vitamin E. The main information collected from all subjects included gender, age, weight and height [W/H ( ≤ 2 years old) or BMI (>2 years old)z-score], clinic features, primary diagnoses categories, and the presence of any chronic disease conditions, infections, and mechanical ventilation (MV). The data of serum D-dimer (DD), blood Lactate (LAC) and procalcitonin (PCT) levels, white blood cell (WBC) and platelet (PLT)count, hemoglobin (HB), albumin (ABL)and triglyceride (TC) concentration were from the patient's routine admission blood test by molecular testing center in our hospital. Mechanical ventilation in PICU was defined as any form of mechanical ventilation for a total of more than 24 h. The severity of illness in the first 12 h was measured by using the pediatric risk of mortality III (PRISM-III) score (19), which was independently evaluated by two attending physicians, and the results were averaged. The cardiovascular sequential organ failure assessment (CV-SOFA) score was used to assess the maximum level of vasopressin use during PICU admission (20), with 0–1: no vasopressors; 2: dopamine ≤ 5 μg/kg/min; 3: dopamine 5–15 μg/kg/min or norepinephrine/epinephrine ≤ 0.1 μg/kg/min; and 4: dopamine >15 μg/kg/min or norepinephrine/epinephrine >0.1 μg/kg/min. The PRISM-III and CV-SOFA scorers were unaware of the vitamin E levels. All patients were followed-up for 30 days, with the 30-day outcome as the endpoint. Patient survival and death were recorded.

To determine the infection status at admission, patients who had any pathogeny microbiology cultured or test on the day of admission to PICU, or who were diagnosed with confirmed or suspected infection within 7 days prior to PICU admission were reviewed by the PICU physician. Confirmed infection was defined as the culture or test of life-threatening pathogenic bacteria, fungal, mycoplasma, or viral pathogen, from the blood, cerebrospinal, pleural, or peritoneal fluid deep sputum (*via* fiber-bronchoscope), or urine (*via* indwelling catheter). Suspected infections included all patients who met the criteria for systemic inflammatory response syndrome, community-acquired pneumonia, or gastroenteritis, but tested negative for microorganisms and received a course of antibiotics. Sepsis, severe sepsis and septic shock were defined according to the International Consensus (21). All data were managed using an electronic data collection form.

### Measurement Method and Range of Reference Values

The serum vitamin E (α-tocopherol) was analyzed using high-performance liquid chromatography–tandem mass spectrometry (22). The pretreatment method of liquid–liquid extraction was used in the project, including a quality control. The experimental standard and internal standard were purchased from Sigma (St. Louis, MO, USA). The quality control was purchased from RECIPE (Munich, Germany). According consensus, serum alpha-tocopherol levels of <5 mg/L are considered vitamin E deficiency (23).

### Statistical Methods

Data were analyzed using SPSS version 20.0 (IBM Corp., Armonk, NY, USA). The Kolmogorov–Smirnov one sample test was used to test whether variables were normally distributed. Data with a no normal distribution were described as the median with 25 and 75% interquartile range (IQR). Categorical variables are presented as counts and percentages. Spearman's correlation coefficients were used to assess the correlation between variables. Mann–Whitney *U*-test for dichotomous variables as most variables were not distributed normally. Frequencies of events were compared using a χ^2^ test. We used univariate and multivariable logistic regressions to assess the influence of risk factors on septic shock. Patient characteristics associated with Alpha-tocopherol in univariate analysis (*P* ≤ 0.10) or potential predictors were included in the multivariable models. *P* < 0.05 in a two-tailed test was taken as criteria for being considered statistically significant. All figures in this study were produced using Medcalc 18.2 software (Ostend, Belgium).

## Results

### Research Profile

**Figure 1:**
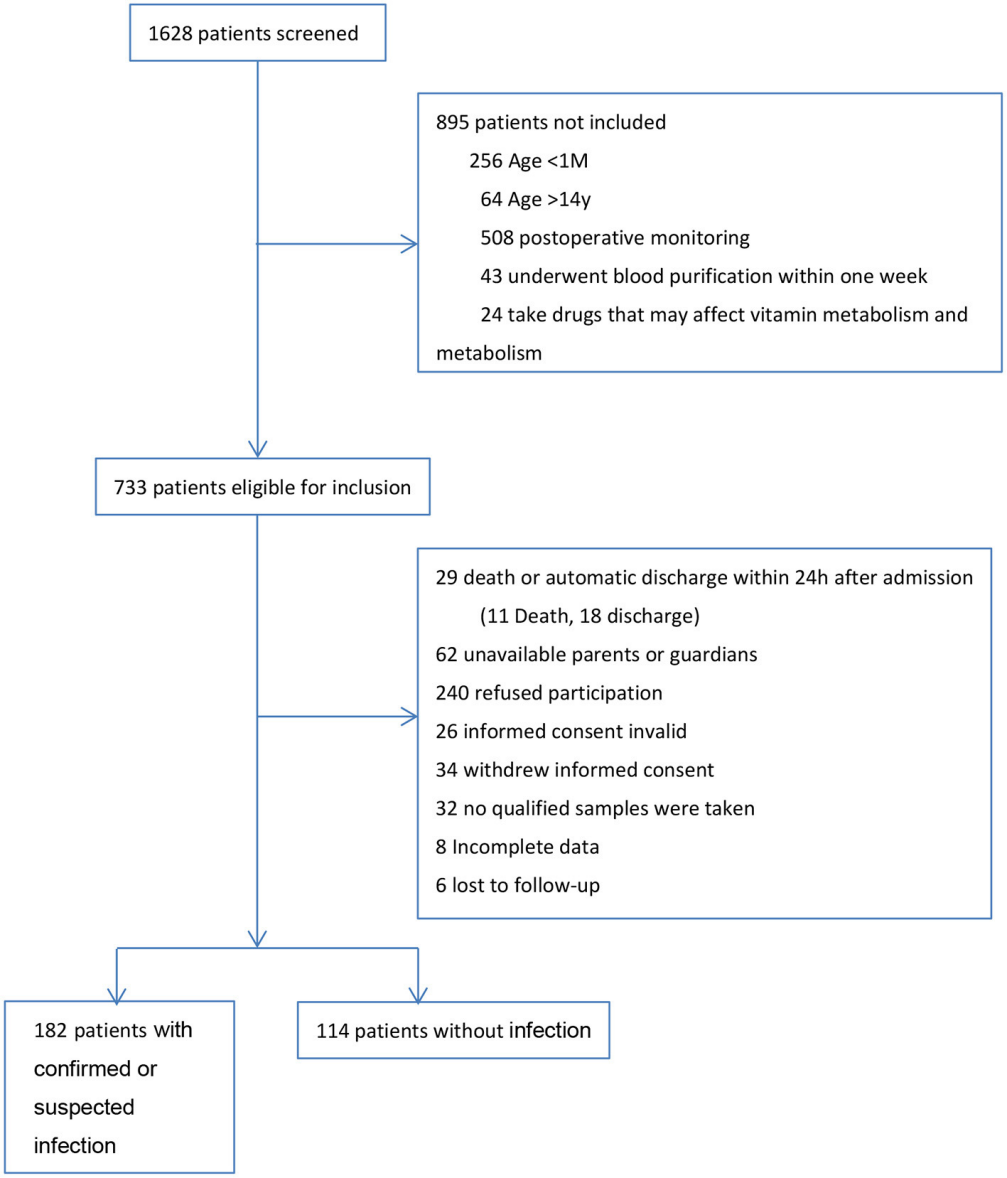
Research profile.

During the 1 year study, we screened all 1,628 patients admitted to PICU. A total of 182 patients with confirmed or suspected infection and 114 patients without infection were included in the final analysis. The research profile and reasons for not enrolling subjects are shown in Figure 1.

### Characteristics of the Study Patients on PICU Admission According to Confirmed or Suspected Infection

The overall cohort had a median serum vitamin E levels of 7 mg/L (IQR 5.1, 8.8) with an age of 20.5 months (7, 56.5) and W/H or BMI z-score of 0.65 (−1.8, 0.5). There was no significant difference in age, gender, BW and CV-SOFA score between patients with confirmed or suspected infection (for short: infection) and non-infection group. The infection group has a lower z-score, higher PRISM-III score, more incidence rate of underlying chronic conditions, mechanical ventilation and mortality. Different from non-infection patients, the most likely primary diagnostic in the infection group was the pulmonary system, followed by the gastrointestinal system.

The mean serum vitamin E status in infection children was significantly lower than that in the non-infection group. Taking 5 mg/L as the cutoff points, the prevalence of vitamin E deficiency was also substantially higher in the infection group. The patients under 60 months of age in the infection group have a significantly lower serum vitamin E levels, and 36.4% children younger than 12 months compared to the non-infection group (Table 1).

**Table 1:** Characteristics of subjects on PICU admission.

	**Confirmed or suspected infection group (*n* = 182)**	**No infection group (*n* = 114)**	***P***
Male (*n*)	110 (60.4%)	61 (53.5%)	0.241
Age (month)	21 (6, 68)	19 (9, 41)	0.874
≤ 12 (*n*)	68 (37.4%)	41 (36%)	0.458
12–60 (*n*)	68 (37.4%)	50 (43.2%)	
>60 (*n*)	46 (25.3%)	23 (20.1%)	
Body weight (kg) (IQR)	11 (7, 14.5)	10.2 (6.5, 16.5)	0.754
*z*-score (W/H, BMI) (IQR)	−0.9 (−2, 0.4)	−0.4 (−1.5, 0.6)	0.012
Z < −2 (*n*)	43 (23.6%)	15 (13.2%)	0.027
Underlying chronic conditions (*n*)	130 (71.4%)	68 (59.6%)	0.036
Mechanical ventilation (*n*)	148 (81.3%)	80 (70.2%)	0.026
CV-SOFA score ≥ 3	42 (23.1%)	34 (29.8%)	0.196
PRISM-III score (IQR)	13.5 (10, 19)	12 (6, 16)	<0.001
30-day mortality (*n*)	33 (18.1%)	6 (5.3%)	0.002
Primary diagnostic categories (*n*)			<0.001
Respiratory	49 (26.9%)	3 (2.6%)	
Neurological	20 (11%)	14 (12.3%)	
Digestive	39 (21.4%)	3 (2.6%)	
Circulatory	12 (6.6%)	51 (44.7%)	
Hematologic, cancer emergencies and others	10 (5.5%)	15 (13.2%)	
Endocrine, immunological and metabolic	25 (13.7%)	13 (11.4%)	
Acute poisoning and trauma	27 (14.8%)	15 (13.2%)	
Mean Vitamin E (mg/L) (IQR)	6.60 (4.7, 8.3)	7.95 (5.9.1, 10.1)	<0.0001
Age, months (IQR)
≤ 12	6.7 (4.9, 8.1)	8.4 (6.8, 10.9)	<0.001
12–60	5.7 (4, 7.6)	6.8 (4.5, 8.9)	0.036
>60	6.3 (4.6, 8.3)	7.8 (5.9, 9.3)	0.080
Vitamin E <5 mg/L (*n*)	55 (30.2%)	17 (14.9%)	<0.001
Age ≤ 12 months (*n*)	20 (36.4%)	3 (17.6%)	0.006

**Figure 2:**
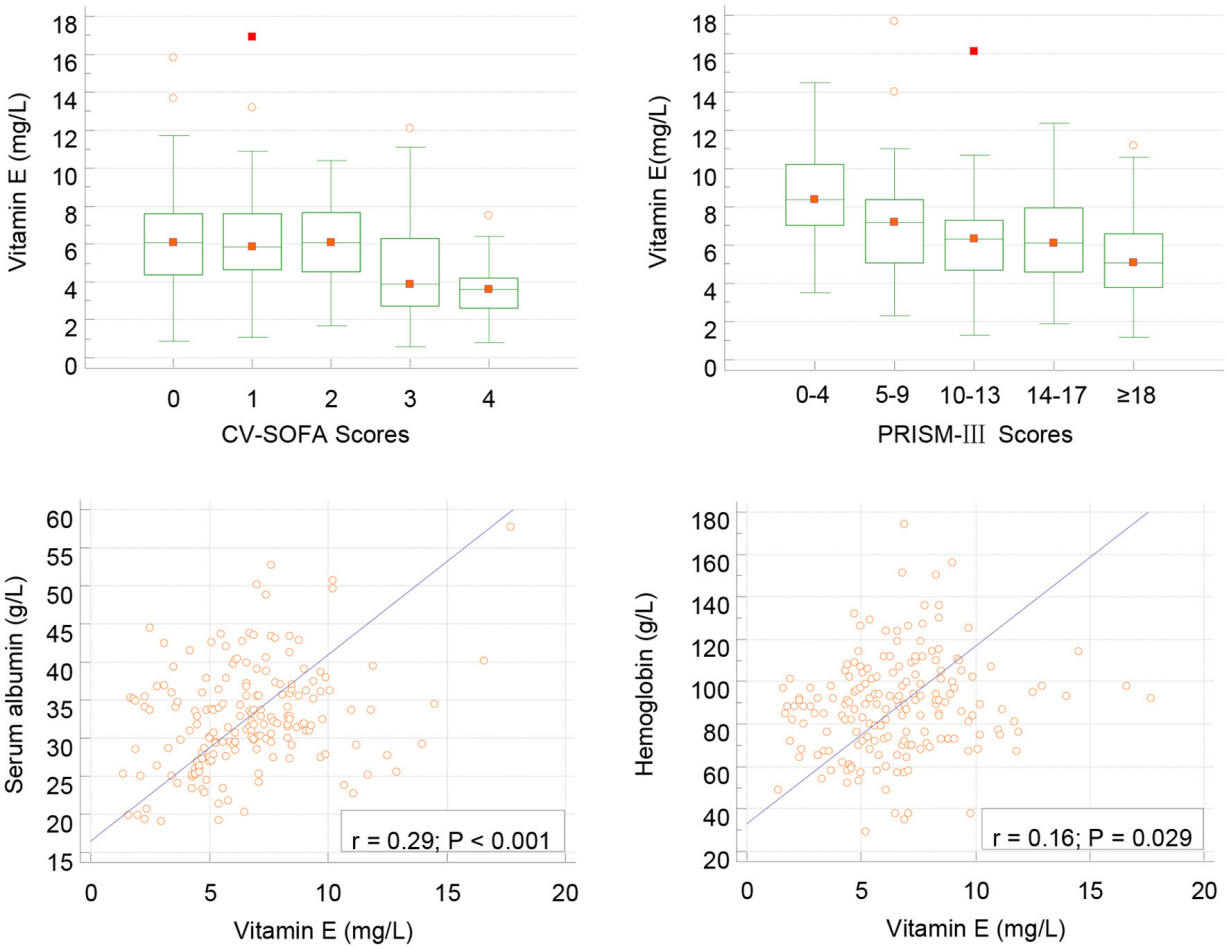
Correlation between serum vitamin E and CV-SOFA (*r* = −0.249, *p* < 0.001), PRISM-III (*r* = −0.238, *P* = 0.001) scores, albumin and hemoglobin levels (Pearson's) in critical children with infection.

In infection group children, lower vitamin E levels were associated with higher PRISMs (*r* = −0.238, *P* = 0.001) and CVSOFA (*r*= − 0.249, *p* < 0.001) scores, and associated with lower albumin and HB levels (Figure 2). We also found that vitamin E levels were related with serum LAC (*r* = −0.275, *P* < 0.001) and DD(*r* = −0.193, *P* = 0.009).

### Clinical Characteristics of Patients With Infection Stratified by Vitamin E Status

The stratified clinical features in the infection group, between patients with and without vitamin E deficiency, showed that there were no significant differences in anthropometry, length of ICU stay, WBC and PLT count. Differences in the ratio of mechanical ventilation, confirmed infection and underlying chronic conditions were also not significant. Compared with infected patients without vitamin E deficiency, those patients with vitamin E deficiency had a higher incidence of sepsis, severe sepsis, septic shock, and 30-days mortality. The PRISM-III and CV-SOFA scores were also higher in patients with vitamin E deficiency. Additionally, compared with infected patients without vitamin E deficiency, patients with vitamin E deficiency had a significantly higher blood PCT, LAC, and DD levels. We also found that patients with vitamin E deficiency had lower ALB counts and HB concentrations than those without vitamin E deficiency (Table 2).

**Table 2:** Clinical features of children with confirmed or suspected infection stratified by vitamin E status.

	**Vitamin E deficiency (*n* = 55)**	**No vitamin E deficiency (*n* = 127)**	***P***
Gender, male (*n*)	32 (58.2%)	78 (61.4%)	0.682
Age (months) (IQR)	28 (7, 92.5)	18 (6, 51.8)	0.169
Weight (Kg) (IQR)	12.0 (7.1, 21.7)	10.5 (6.7, 14.5)	0.294
*Z*-score (IQR)	−0.9 (−1.97, 0)	−1.0 (−2, 0.5)	0.586
Confirmed infection (*n*)	23 (41.8%)	64 (50.4%)	0.288
Sepsis (*n*)	43 (78.2%)	67 (52.8%)	0.001
Severe sepsis (*n*)	33 (60%)	35 (27.6%)	<0.001
Septic shock (*n*)	26 (47.3%)	16 (12.6)%	<0.001
PLT (×10^∧^9/L) (IQR)	188 (129, 306)	217 (133, 275)	0.692
PRISM-III (IQR)	15.7 (6.32)	12.9 (6.41)	0.007
PCT (μg/L) (IQR)	12.3 (10.4, 14.4)	9.1 (4.2, 14.0)	0.014
WBC (×10^∧^9/L) (IQR)	8.67 (6.2, 14.3)	9.4 (6.5, 14)	0.784
Lactate (mmol/L) (IQR)	1.6 (1.0, 3.7)	1.2 (0.8, 2.1)	0.023
MV (*n*)	48 (87.3%)	100 (78.7%)	0.176
Length of ICU stay (days) (IQR)	6 (3, 9)	6 (4, 9)	0.961
30-day mortality (*n*)	15 (27.3%)	18 (14.2%)	0.035
CV-SOFA (IQR)	2 (1, 3)	1 (0, 2)	<0.001
≥3 (*n*)	26 (47.3%)	16 (12.6%)	<0.001
Underlying chronic condition (*n*)	43 (78.2%)	87 (68.5%)	0.185
DD (mg/L) (IQR)	3.21 (1.07, 12.64)	1, 27 (0.55, 3.95)	0.001
ALB (g/L) (IQR)	28.5 (25.1, 34.5)	32.9 (29.5, 37.3)	<0.001
HB (g/L) (IQR)	85 (67, 95.8)	90 (73, 104)	0.043

*Testing for association with serum 25(OH)D level by Mann–Whitney test (dichotomy) or chi-square test*.

### Comparison of Septic and Non-septic Shock Patients in Confirmed or Suspected Critically Ill Children

In total, 42 patients (23.1%) in the infection group suffered from septic shock. The comparisons between patients with and without septic shock in infection patients are presented in Table 3. The distributions of age, gender, weight, and z-score were similar in the two subgroups.

**Table 3:** Comparison of septic and non-septic shock patients.

	**Sepsis shock (*n* = 42)**	**No sepsis shock (*n* = 140)**	***P***
Age (months)	22.5 (5, 54)	21 (6.5, 74.5)	0.597
Weight (Kg)	11 (6.5, 16.6)	11.3 (8, 14.3)	0.787
Male (*n*)	27 (64.3%)	83 (59.3%)	0.562
*z*-score (W/H, BMI)	−1.3 (−2.2, 0)	−0.9 (−1.3, 0.5)	0.085
Underlying chronic conditions (*n*)	28 (66.7%)	102 (72.8%)	0.436
Mechanical ventilation (*n*)	37	111	0.199
Confirmed infection (*n*)	18 (42.9%)	57 (40.7%)	0.805
CV-SOFA score	3 (3, 4)	1 (0, 1)	<0.001
PRISM-III score	18 (15, 21)	13 (8, 17)	<0.001
≥10 (*n*)	40 (95.2%)	101 (72.1%)	<0.001
30-days mortality (*n*)	16 (38.1%)	17 (12.1%)	<0.001
Mean Vitamin E (mg/L)	4.6 (3.4, 7)	6.8 (5.3, 8.4)	<0.001
Vitamin E <5 mg/L (*n*)	26 (61.9%)	29 (20.7%)	<0.001
PICU Length of stay, (days)	6 (3, 9)	6 (4, 9)	0.783
DD (mg/L)	2.32 (0.86, 15.9)	1.28 (0.61, 3.58)	0.012
PLT (×10^∧^12/L)	187.5 (122, 269)	219.5 (135, 305)	0.215
PCT (μg/L)	12.9 (5.2, 18.0)	10.1 (4.5, 14.5)	0.122
WBC (×10^∧^9/L)	8.9 (6.1, 16.9)	9.4 (6.4, 13.7)	0.968
LAC (mmol/L)	3.5 (1.3, 7.7)	1.1 (0.8, 1.8)	<0.001
HB (g/L)	84.5 (68.4, 89.6)	89.5 (74, 104)	0.012
ALB (g/L)	28 (25.3, 34.9)	32.6 (29.4, 37)	0.004

*Testing for association with serum 25(OH)D level by Mann–Whitney test (dichotomy) or chi-square test*.

The lower median serum level of vitamin E and the incidence of vitamin E deficiency (<5 mg/L) were significantly higher in patients with septic shock than in those without septic shock. Children with septic shock had a higher median of PRISM-III and CV-SOFA scores. Lactate and DD levels were also higher, but ALB and HB levels were lower in the septic shock group. Children with septic shock have higher 30-days mortality. While there was no significant statistical difference in the ratios of mechanical ventilation, confirmed infectious and underlying chronic conditions between the two groups. There were also no significant differences in WBC and PLT count, PCT levels and Length of ICU stay.

### Regression Analysis to Identify Predictors of Septic Shock

Univariate analysis was performed to compare the patients with and without septic shock in the infection patients group, variables with *P* < 0.10 were considered as significant contributors to septic shock (Table 4).

**Table 4:** Logistic regression models assessing the variables associated with septic shock.

	**OR**	**95% C.I**.	**Sig**.
**Univariate analysis**			
Age, per 1 month increase	0.997	0.989–1.005	0.461
Weight, per 1 Kg increase	0.985	0.940–1.031	0.505
Male vs. Femal	0.809	0.396–1.655	0.561
Z-score (W/H, BMI) < −2 vs. >−2	1.644	0.762–3.545	0.205
Underlying chronic conditions, yes vs. no	0.745	0.355–1.565	0.437
PRISM-III score, per 1 U increase	1.144	1.073–1.221	0.000
Vitamin E, per 1 mg/L decrease	1.317	1.124–1.542	0.001
Vitamin E <5 mg/L, yes vs. no	6.220	2.953–13.101	0.001
Confirmed infection, yes vs. no	0.991	0.497–1.976	0.978
DD, per 1 U increase	1.023	0.999–1.047	0.065
PCT, per 1 μg/L increase	1.032	1.004–1.060	0.027
LAC, per 1 mmol/L increase	1.806	1.422–2.293	0.000
ALB, per 1 g/L increase	0.932	0.880–0.987	0.016
HB, per 1g/L increase	0.980	0.964–0.996	0.013
**Multivariable**			
Age, per 1 month increase	0.984	0.946–1.023	0.413
Weight, per 1 Kg increase	1.027	0.396–2.664	0.956
Male vs. Femal	1.050	0.842–1.309	0.667
Z-score (W/H, BMI) < −2 vs. >−2	1.290	0.419–3.978	0.657
PRISM-III score, per 1 U increase	1.101	1.015–1.195	0.021
Vitamin E, per 1 mg/L decrease	1.250	1.016–1.537	0.034
Vitamin E <5 mg/L, yes vs. no	6.749	2.449–18.60	<0.001
DD, per 1 U increase	1.024	0.996–1.052	0.094
PCT, per 1 μg/L increase	1.026	0.991–1.061	0.145
LAC, per 1 mmol/L increase	1.894	1.407–2.549	0.001
ALB, per 1 g/L increase	1.000	0.928–1.077	0.996
HB, per 1 g/L increase	0.0986	0.964–1.009	0.238

Based on the statistically significant differences results in the univariate analysis, the multivariable regression analysis indicated that PRISM-III score, vitamin E, DD and LAC levels were associated with septic shock (Table 4). An independent impact of vitamin E deficiency on septic shock was identified.

Serum TG was measured in 77 sepsis patients at PICU admission, and the mean levels in septic shock (0.99 ± 0.38, *n* = 18) showed no statistically significant differences with the non- septic shock group (0.94 ± 0.31, *n* = 59, *P* = 0.528). TG level was not correlated with vitamin E (*r* = −0.1909, *P* = 0.0963). However, no patients had clinically high TG levels (>1.8 mmol/L). The addition of this group did not affect the regression models for septic shock.

## Discussion

In this prospective cohort study, we found a high incidence of vitamin E deficiency in critically ill children with confirmed or suspected infection aged 1 month to 14 years (30.2%), especially within sepsis or septic shock (61.9%). The majority of patients were younger than 12 months age, with lower W/H or BMI z-score and/or often underlying chronic conditions. That means most of these patients were malnutrition. The overall mortality of patients with infection in 30 days after PICU admission was 18.1% in the study. For infectious children with vitamin E deficiency, 30 days mortality after PICU admission was 27.3%.

The long-term association between vitamin E status and overall mortality in critically ill children has not been sufficiently studied and analyzed. The research suggested that lower vitamin E concentrations were inversely related to total mortality, and show that vitamin E adipose tissue stores may be depleted after burn injury in pediatric patients (24).

Our study demonstrated significantly higher morbidity of vitamin E deficiency in severe sepsis and septic shock than other children with confirmed or suspected infection in the PICU. Remarkably, we revealed that patients with vitamin E deficiency were more likely to suffer severe sepsis and septic shock, which can directly result in adverse outcomes. Our other findings of this study were serum PCT, LAC, DD levels in vitamin E deficiency patients markedly increased and reversely corrected with vitamin E levels.

In recent years, a growing body of research has shown that pathophysiological changes in patients with sepsis was associated with oxidative stress and inflammatory response, which happens when reactive oxygen species production and the antioxidant protection mechanisms, as well pro-inflammatory and anti-inflammatory mediators are imbalanced, leading to pathological injury, organ dysfunction and septic shock, this provides a rationale to support a potential therapeutic role for antioxidant and anti-inflammatory therapy (4, 5).

Vitamin E is a potent antioxidant and anti-inflammatory that can protect the stability of cell membranes by disrupting the chain reaction caused by free radicals, and regulate the body's metabolic activities in an orderly manner (25). A systematic analysis of the relationship between vitamin E and septic shock has not been previously conducted. We found that the incidences of 30-days mortality in children with vitamin E deficiency were higher than that in those patients without vitamin E deficiency. Patients with septic shock had significantly lower levels of vitamin E, which was closely related to higher PRISM-III and CV-SOFA scores, which suggests that low serum levels of vitamin E increased adverse outcomes.

Malnutrition is a widespread problem among children with infection, the low vitamin E levels in sepsis patients may be ascribed to low dietary vitamin E intakes and small adipose tissue reserve of vitamin E. Therefore, when the disease continues to persist, the prevalence of vitamin E deficiency in children with sepsis and septic shock may be higher than that shown in the present study. For another, increased catabolism, a large consumption by oxidative stress and inflammatory responses also reduce the level of vitamin E. Thus, in the situation of vitamin E deficiency, the oxidative stress and inflammation response were more aggravated, which are the adverse conditions in the early stage of sepsis. It is conceivable that vitamin E deficiency may partially contribute to the hyperinflammatory responses and Oxidative stress in sepsis because of the importance of vitamin E in antioxidant protection and anti-inflammatory immunity.

In addition, our data indicated that there is a statistical correlation in vitamin E and albumin and HB levels in children with sepsis and septic shock. As the most abundant circulating protein in the plasma, human serum albumin also exerts important antioxidant activities against oxidative damage (26). Children with sepsis can consume a lot of albumins; this is consistent with vitamin E consumption in sepsis patients. Vitamin E deficiency can shorten the lifespan of erythrocyte likely because of increased oxidant stress and antioxidant consumption (13), which may be one of the reasons why sepsis children are prone to anemia.

Remarkably, by evaluating the relationship using regression approaches, a statistically significant inverse relationship between vitamin E levels and sepsis shock was reported. Furthermore, after variable adjustment by the multivariable logistic model, vitamin E deficiency was revealed to be independently associated with septic shock. Lower vitamin E levels are likely to be linked to disease severity and may assist help in evaluating the progression and prognosis of sepsis children.

As we all know, it is difficult to prove the adverse consequences of vitamin E deficiency on humans. Although vitamin E deficiency has been an issue of concern for decades in the general population, there is still a need for in-depth and extensive research with regard to vitamin E status in human's health benefits. Usually, in healthy children or children with common diseases, low serum levels of vitamin E may not cause noticeable symptoms or may manifest as mild neurological abnormalities (18). Symptoms of chronic and severe vitamin E deficiency in humans include progressive neurological disorders, spinocerebellar ataxia, which is the result of the death of peripheral nerves, especially sensory neurons (24). But its specific role is extremely unclear in children with critically ill or severe sepsis.

For another, it is also challenging to assess the status of vitamin E because it is fat-soluble; therefore, low serum vitamin E levels could reflect a variety of factors, including low circulating lipid concentrations, low vitamin E intakes, or high oxidative stress and inflammatory response levels. Besides, serum vitamin E concentrations are also associated the transport and delivery by lipoproteins and lipids (27). Thus, alterations in lipid metabolism could alter vitamin E status. Although study suggested that vitamin E have negative correlation with triglyceride in both normal and abnormal patients (28), however, according to the triglyceride statuses were tested in 77 patient in this study, we did not find children with hypertriglyceridemia, and no correlation was found with serum vitamin E level, we also did not find significant difference of triglyceride levels between sepsis and no-sepsis children.

In this study, because all patients were recruited from one medical center, the restricted number of sepsis children limited the generalizability of the data. Additionally, the details about the underlying diagnoses of the non-infection group were not collected, so whether there was a wide-spread immune activation in these patients was not clear. And vitamin E levels were evaluated only one time upon PICU admission; thus, the variations in vitamin E status during the PICU period were largely unknown. However, given the well-appreciated malnutrition, hypermetabolic and profoundly catabolic state and disordered oxidative stress and inflammatory response, the depletion and deficiency of vitamin E may be a very significant problem among children with severe sepsis and septic shock.

The next step is to consider what effect additional vitamin E supplementation may exert on ameliorating the oxidative stress and inflammatory response of sepsis and septic shock children. Studies have found that in adult patients with sepsis, early enteral pharmaconutrition with vitamin E in combination with an immunonutrition formula results in significantly faster recovery of organ function compared with control (29). But other literature also shows that the absence of altered redox settings in the blood of neonates during sepsis episode, and vitamin E-provoked decrease in the activity of some components of AOS, suggest that the supplementation of vitamin E in neonates might not be rational (30). However, our study focused on pediatric patients but did not include neonates, so the roles of supplementation of vitamin E in pediatric patients aged from 1 month to 14 years old with sepsis and septic shock remains unclear or controversial and need further study. Additionally, both the lack of an IV vitamin E supplement and the difficulty in perioral vitamin E use during sepsis and septic shock, which may lead to impaired drug absorption, present huge challenges (31). As a result, more well-controlled, randomized, prospective studies are needed to document its protective effect.

## Data Availability Statement

The raw data supporting the conclusions of this article will be made available by the authors, without undue reservation.

## Ethics Statement

The studies involving human participants were reviewed and approved by Institutional Review Board of Children's Hospital of Chongqing Medical University. Written informed consent to participate in this study was provided by the participants' legal guardian/next of kin.

## Author Contributions

HD and FX: conceptualisation, writing-review, and editing. JL: data curation and investigation. HD: formal analysis, methodology, software, and writing-original draft. FX: project administration and supervision. CL: resources and visualization. JL, CL, and FX: validation. All authors contributed to the article and approved the submitted version.

## Conflict of Interest

The authors declare that the research was conducted in the absence of any commercial or financial relationships that could be construed as a potential conflict of interest.
